# Understanding Design Features of Music and Language: The Choric/Dialogic Distinction

**DOI:** 10.3389/fpsyg.2022.786899

**Published:** 2022-04-22

**Authors:** Felix Haiduk, W. Tecumseh Fitch

**Affiliations:** ^1^Department of Behavioral and Cognitive Biology, University of Vienna, Vienna, Austria; ^2^Vienna Cognitive Science Hub, University of Vienna, Vienna, Austria

**Keywords:** language, music, information theory, choric, dialogic, animal communication

## Abstract

Music and spoken language share certain characteristics: both consist of sequences of acoustic elements that are combinatorically combined, and these elements partition the same continuous acoustic dimensions (frequency, formant space and duration). However, the resulting categories differ sharply: scale tones and note durations of small integer ratios appear in music, while speech uses phonemes, lexical tone, and non-isochronous durations. Why did music and language diverge into the two systems we have today, differing in these specific features? We propose a framework based on information theory and a reverse-engineering perspective, suggesting that design features of music and language are a response to their differential deployment along three different continuous dimensions. These include the familiar propositional-aesthetic (‘goal’) and repetitive-novel (‘novelty’) dimensions, and a dialogic-choric (‘interactivity’) dimension that is our focus here. Specifically, we hypothesize that music exhibits specializations enhancing coherent production by several individuals concurrently—the ‘choric’ context. In contrast, language is specialized for exchange in tightly coordinated turn-taking—‘dialogic’ contexts. We examine the evidence for our framework, both from humans and non-human animals, and conclude that many proposed design features of music and language follow naturally from their use in distinct dialogic and choric communicative contexts. Furthermore, the hybrid nature of intermediate systems like poetry, chant, or solo lament follows from their deployment in the less typical interactive context.

## Introduction

Music and language are two human cognitive and communicative systems that are similar in a variety of ways: the vocal-auditory domain is typically the primary modality, but it is not the only one (writing, sign, or dance are others). Both utilise the same vocal apparatus, and similar motor systems and perceptual physiology. Their respective neural underpinnings have major shared portions. Both consist of elements combined in a hierarchical manner by certain, culture-specific rules. Both systems are learned, but have biological components shared with other species. Despite these many similarities, this paper is concerned with the differences between the two systems. Why should two universal human systems, that share so much, nonetheless exhibit consistent differences?

It is clear that there is a great variety of music and language within and across cultures, and what is termed ‘music’ varies within a culture (see Trehub et al., 2015; Thompson et al., 2019), fulfilling a broad range of psychological purposes that influence their acoustic features. For example, while dance music will engage motor systems, lullabies are used for soothing infants, and this translates into consistent acoustic differences cross-culturally (Mehr et al., 2018). Similarly, language changes when playing with young infants, reciting a mantra in a ritual, or engaging in political discussions. However, despite this variety, certain features seem to differentiate many instances of music and language (which we will term ‘typical’ in this paper). Hockett (1960) and Fitch (2006) termed these prototypical properties ‘design features’ of language and music, respectively (see Table 1). Nonetheless, borders between language and music are not clear cut (as in the case of poetry or religious chanting), and particular instantiations of music and language can be ‘more musical’ or ‘more linguistic’ than prototypical instances.

**Table 1:** Design features differing between language and music, updated from Fitch (2006).

Design Feature	Language		Music		Definition
	**V**	**S**	**V**	**I**	
Vocal auditory channel	+	−	+	−	Signal sequences are patterns of sounds produced by the vocal tract and articulators
Broadcast transmission	+	+?	+	+	Signal sequences are detectable by anyone within given distance/line of sight
Rapid fading	+	+	+	+	Signal sequences dissipate when signalling stops
Interchangeability	+	+	+	−	Individuals can be both sender and receiver
Total feedback	+	+^1^	+	+?^1^	Senders themselves perceive what they signal
Specialisation	+	+	+	+	A signal sequence does not directly trigger a specific behaviour in the receiver
Productivity	+	+	+	+	Ability to produce novel signal sequences
Discreteness	+	+	+	+	Signalling units are functionally distinct
Cultural transmission	+	+	+	+	The signalling system is transmitted between individuals *via* learning and teaching
Movement^2^	+	+	+	+	Movements of body (−parts) accompany movements that create the signal itself
Transposability	+	+	+	+	The relationships between signal units rather than absolute features identify a signal sequence (a sentence is considered the same regardless of who spoke/signed it, a melody regardless of instrument, voice or absolute pitch)
Duality of Patterning	+	+	−	−	Signal sequences can be analysed both as units of signalling (cenemes) and meaning-bearing units (pleremes)
Generativity	+	+	+	+	Signal units are recombined according to rules
Semanticity	+	+	−	−	Fixed associations exist between meaning-bearing units and states or properties of the world/environment
Arbitrariness	+	+	−	−	The content of most meaning-bearing units is unrelated to features of signalling units
Displacement	+	+	−	−	Meaning-bearing units refer to entities outside their spatial and temporal context
Discrete pitches	−	−	+	+	Allowed pitches are based on a scale of tones related by intervals
Isochronic	−	−	+	+	Regular periodic pulse providing a reference framework for other temporal features of the signal sequence
Performative context	−	−	+	+	Classes of signal sequences (e.g. songs or styles) recur in specific social contexts
Repeatable (repertoire)	−	−	+	+	Signal sequences are distinguishable (pieces), exactly repeatable and repeated in certain contexts
A-referentially expressive	−	+?	+	+	Higher order relations of a signal sequence are cognitively mapped to movement and affective responses

*These design features concern speech (including sign) or musical acts that we label as ‘typical’, e.g., spoken conversations or musical ensemble playing. V = vocal, S = signed, I = instrumental*.

1 *Sensorimotor*.

2 *Added by the authors*.

In this paper, we propose a framework that aims to explain design features differentiating music and language as responses along three continuous dimensions. (1) the **goal** of the linguistic or musical act, with a more propositional or more aesthetic focus; (2) the repetitiveness or **novelty** of the events within a linguistic or musical sequence and (3) crucially, the **interaction** and temporal coordination between individuals participating in linguistic or musical acts, the poles of which we term ‘choric’ and ‘dialogic’. While the first two dimensions are widely recognised and discussed in comparisons of music and language, the last one has more often been neglected. We think that the interaction dimension is a crucial addition for understanding design feature differences between language and music, because major acoustic differences between spoken language and music are rooted in social cognition and interaction.

We derive these dimensions by applying a ‘reverse-engineering’ approach, based on information theory, starting from the observed design features. This framework supports predictions about the changes in design features expected for ‘nontypical’ instances of music and language, thus laying the foundations for a more fine-grained and continuous analysis of music and language when used for different psychological and social purposes.

Our comparison of music and language focusses on social interactions and starts from the auditory domain, based on the premise that written communication is a derived form. However, both systems go beyond the purely acoustic domain (Cross et al., 2013; Levinson and Holler, 2014; Honing, 2018). For example, both music and language incorporate body movement in the form of dance and co-speech gestures, or mime and sign languages (which are typically silent). Although our framework takes the auditory domain as a starting point, we expect that it can also be applied more generally to movement-based communication, predicting changes in movement-based communication and incorporating movement into speech or song. We thus think our framework might also be useful for analysing animal communication, both acoustic and multimodal.

Our framework is in principle compatible with various hypotheses about the evolutionary relation of language and music (see Cross et al., 2013). We assume only that variation in acoustics occurs based on social and perceptual goals, pointing at fundamental relevant traits, but remain agnostic with regards to the evolutionary processes involved (biological and/or cultural) and/or the origin states of language and music (e.g., from a common audio-vocal precursor system, as Darwin, 1871 proposed). However, our framework does assume a pivotal role for audio-vocal communication at some point in evolution, thus incorporating the phylogenetically unusual trait of vocal learning (Fitch, 2006; Jarvis, 2019), which is shared by both systems. Crucially, our framework avoids dichotomous conceptions of music and language as either fully distinct or fully indissociable faculties. This notion of the differences along a continuum follows naturally both from neural evidence and from the existence of styles intermediate between music and language (poetry, rap, lament and others).

The paper is structured as follows: the first section presents the conceptual and theoretical foundations for our framework: a reverse engineering approach allows us to derive three dimensions from design features differing between language and music. The three dimensions described—goal, novelty, and interactivity—create a space within which both prototypical and non-canonical forms of both music and language can be situated. Information theory makes these design features predictable. The three sections that follow discuss the three dimensions in more detail, arguing that the characteristic design features of music and language can be understood as a function of their deployment within this three-dimensional space. The last section opens the door to comparative cognition, arguing that some vocal communication in non-human animals can also be fruitfully understood using our framework, and ends with predictions and suggestions for questions to be addressed in future empirical research.

## Conceptual and Theoretical Framework

### Design Stance and Reverse Engineering

In addition to investigations of neural and cognitive processes, individual development and cultural specifics, a deeper understanding of both language and music and how they differ requires inquiry into their evolutionary origin(s). Various hypotheses have been proposed regarding the origin of music, often concerned with finding an adaptive value (see Mehr et al., 2021; Savage et al., 2021 for recent reviews of the debate). We will not focus on possible adaptive values in this paper, nor will we investigate the causal roles of the many possible evolutionary, cultural or developmental processes involved. Rather, we will take a design stance and a ‘reverse engineering’ approach, using the design features proposed by Hockett (1960) and Fitch (2006; see Table 1) as a starting point for our framework.

The ‘design’ stance has a long tradition in biology and relies on the idea that under certain constraints evolutionary processes act to refine and optimize traits as would an engineer (Hockett, 1960; Krebs and Davies, 1997; Maynard Smith, 2000; Csete and Doyle, 2002; Richardson, 2003; Tooby and Cosmides, 2005); the use of ‘design’ in this context implies natural selection and has no association with unscientific notions of ‘intelligent design’. This allows us to ask what constraints on concrete linguistic or musical acts could plausibly yield the observed design features differentiating music and language. We will conceptualise these constraints as poles of continuous dimensions, creating a multidimensional conceptual space. Crucially, this continuous space allows us to predict how design features of non-typical instances of language and music, such as poetry or rap, should vary as a response of their deployment along the dimensions proposed.

First, note that the kind of elements that make up language and music differ. Language consists of phonemes that are the building blocks for meaning-bearing units like morphemes and words, which in turn are combined to yield sentences. This organisation rests on the need to convey propositional meaning, which is a key characteristic of prototypical language use, but not of prototypical music. Accordingly, the design feature of semanticity and those derived from it (arbitrariness, displacement and duality of patterning) discriminate prototypical language from prototypical music. Although sung music that uses lyrics is common, there is no requirement to perceive lyrics in order to recognise a sound sequence as music, and much music is purely instrumental. Music instead has stronger links to movement, and to emotional and aesthetic appraisal (Huron, 2006; see also Thompson et al., 2019 for cross-cultural perspectives). Fitch (2006) subsumes the expressive mappings of musical form to movement and emotions under the design feature ‘a-referentially expressive’.

These contrasting design feature differences between prototypical language and music suggest a trade-off between a primary goal of conveying semantic meaning for language (which we term ‘propositional’) and a goal of aesthetic appraisal (in a broad sense, see Huron, 2016) for music. We suggest that many observed design feature differences can be explained by interlocutors following either aesthetic or propositional goals. Notably, both aesthetic and propositional goals require predictive cognitive processes, but in different ways, as reviewed below. But simply categorizing music as aesthetic and language as propositional is also incomplete—some ways of speaking also pursue aesthetic goals, as in poetry, while some music has propositionality, like humming ‘Happy Birthday’, to indicate gift-giving, or songs mimicking birdsong. It is thus useful to conceive of music and language as lying on a propositional-aesthetic continuum, where language typically tends towards the propositional side while music tends towards the aesthetic side, but with some instances between these poles. We will term this continuous axis the ‘**goal**’ dimension.

A second dimension further partially differentiates language and music. Conversational language typically conveys a large amount of novel semantic information (Grice, 1975) and exact repetition is unusual. Music in contrast is typically characterised by repetition at multiple levels, from single tones or chords, motifs, and melodies, up to repeated performances of entire musical pieces. This is supported by two further contrasting design features: while language has gliding intonation, flexible lexical tone and continuously variable syllable durations, music typically consists of tones of fixed pitches organised in scales, and is prototypically characterised by rigorous timing based on isochronous meter (for exceptions see Savage et al., 2015). Thus, in music, both the temporal and the spectral acoustic dimensions relate their elements by small integer ratios. Repeatability is further related to the design feature of performative context, where certain kinds of music are repeated in specific cultural situations (e.g., lullabies to soothing babies). Repeating the same phrases does occur in language, but mostly in specific cultural situations like religious or artistic acts (e.g., prayers or poems). Typically, however, repetition is uncharacteristic of everyday conversations but abundant in music making (Savage et al., 2015). This repetitive-novel continuum is thus another dimension where music and language have a different focus, although again certain instances occupy the middle ground along the continuum. We will call this the ‘**novelty**’ dimension.

Both the goal and the novelty dimensions are widely known and discussed, and both involve predictive cognitive processes. However, although language and music can be deployed at several points along these dimensions, the predictive cues they provide differ (e.g., music has a much smaller set of possible temporal and frequency constituents than speech). We will argue that these differences make sense only when a third dimension is added, involving the timing of individual performances in a dyad or group. As Brown (2007) has argued, an important difference between music and language is their temporal coordination. Language prototypically exhibits sequential turn-taking, where speakers typically have little overlap in their utterances. In music, simultaneity is both possible and typical: music is often performed by several people simultaneously. We will adapt the term ‘concurrent’ to refer to individuals simultaneously performing (vocalising, playing), specifically when these signals are coupled (causally related) and coordinated (thus excluding two unconnected conversations at the same party). Concurrence does not necessarily imply the same events happening at precisely the same time (which we term ‘synchronous’, following Ravignani et al., 2014). We dub the end of this dimension that involves turn-taking and alternation ‘**dialogic**’, and the pole featuring concurrent performance ‘**choric**’ (from the Greek *choros* meaning ‘chorus’). We choose these novel terms to specifically imply joint action: deliberate coordination within a common representational framework (see Sebanz and Knoblich, 2009, 2021; for music, e.g., Keller et al., 2014; for language, e.g., Tomasello, 2010). While turn-taking requires cues to predict the end of the current speaker’s phrase, concurrence requires much more fine-grained ongoing predictions about subsequent events in a vocal sequence. Again, this **choric/dialogic axis** defines a continuum, and there are intermediate cases of dialogic form in music, for example exchanging solos in jazz or call-and-response songs, and concurrence in language, such as group chanting or recitation. We call this axis the ‘**interactivity**’ dimension.

The purpose of these three dimensions (see Table 2) is to conceptualise a continuous space that can account for both prototypical instances of language and music and instances that are not considered typical, and to explain their design features as a consequence of the deployment along these dimensions.

**Table 2:** Overview of the three proposed dimensions of our framework, with examples from music, language, and animal communication.

Dimension	Pole 1		Pole 2	
	Name	Example	Name	Example
Goal	Propositional	Discussing the week’s events with a friend Singing ‘Happy Birthday’	Aesthetic	Shakespeare sonnetsListening to your favourite Beatles album
Novelty	Novelty	Listening to a conference talkVariation and recombination of melodic modules in BaAka music (Lewis, 2021)	Repetition	Word repetition for emphasis (‘I did not break the dish. I did not break the dish. I repeat, I did not break the dish’)Choruses in songs
Interactivity	Choric	Religious ensemble chantingEnsemble musicPlain-tailed wren mating display (within sex)	Dialogic	Conversational speechCall-and-response songAnimal antiphonal calling

Hockett’s design stance as applied to language has been criticised for neglecting cognition, being biased concerning the modality of transmission (auditory-vocal) and focussing on surface aspects of the linguistic code rather than its content (Wacewicz and Żywiczyński, 2015). However, these criticisms are less telling regarding music, and our approach attempts to overcome any such limitations. For example, we start our comparison of language and music assuming an auditory-vocal modality, but emphasize that it can also be applied to signed languages or mime, and incorporate facial expressions, gestures and body language, as long as information trajectories can be measured in the target domain (see Table 3). Crucially, cognition plays a central role in our framework, *via* the mutually predictive role of the participants in temporally unfolding musical and linguistic acts, which require complex multi-time scale cognitive processes.

**Table 3:** Assumptions and measures of information theory.

Assumption	Measure/method	References
Information is an adequate model of prediction, plausible to happen in the brain	Predictive coding and similar accounts	Friston, 2010; McDonnell et al., 2011; Pearce and Wiggins, 2012; Crupi et al., 2018; Koelsch et al., 2019
Entropy and information can be measured at multiple levels of the signal sequence concurrently, and their interaction can be modelled	Models based on statistical learning and using a multiple viewpoint approach	Pearce and Wiggins, 2012 Forth et al., 2016 see also Rohrmeier and Koelsch, 2012
The information/entropy trajectories of the different levels can be compared	Mutual information measures for multivariate time series (transfer entropy, partial information decomposition, etc.)	Hlaváčková-Schindler et al., 2007; Williams and Beer, 2010 (preprint); Williams and Beer, 2011 (preprint)
Context (e.g., discourse context, conceptual knowledge, etc.) can be modelled using information theory	Conditional entropy (e.g., with *n*-gram models)	Piantadosi et al., 2012 Mahowald et al., 2013 see also Kuperberg and Jaeger, 2016; see also Venhuizen et al., 2019
Information theory can be applied to both discrete or continuous (or discretisable) sequences, e.g., for body movement and gesturing	Discretisation of continuous signals Sample entropy, multiscale entropy	Glowinski et al., 2013; Zbili and Rama, 2021 Glowinski et al., 2010; Glowinski and Mancini, 2011

### Information Theory

All three of our dimensions centrally involve predictive processes. In language (goal dimension), inferring propositional meanings involves prediction at the level of semantics, while aesthetic experiences exploit the interplay between fulfilment of expectations and deviation from predictions. Repetition entails high predictability, while novelty implies low predictability. Finally, for the interactivity dimension, coordinating events in time between several individuals requires either prediction or reaction, with prediction being faster and more flexible.

Prediction involves estimates of probability: at any given point during the musical or linguistic act, possible subsequent events are assigned a probability given the current context, influencing the perceiver’s expectations about what happens next. Predictions always have a degree of uncertainty, allowing some possibility of other events to occur instead (even a highly familiar event may be corrupted by noise or mistakes). Thus, in order to support successful prediction, a signaller should decrease the uncertainty about subsequent events. Signals can in this way be analysed in terms of the change in their information over time.

The scientific field dealing with reduction in uncertainty is information theory, originally formulated by Shannon (1948), and we will use information theory as our theoretical foundation when analysing the deployment of language and music along the goal, novelty, and interactivity dimensions. The common currency is information, which is simply reduction in uncertainty, quantified in bits. If an event in a sequence is highly predictable, that event’s information content—should it occur—is low. Unexpected events are surprising and have a high information content, hence information content is also termed ‘surprisal’. Information theory has developed considerably since Shannon’s fundamental insights, and now provides a rich toolbox for analysing a variety of phenomena (see Table 3; Crupi et al., 2018). In a crucial addition, the uncertainty of predictions themselves, i.e., the confidence in or precision of one’s own predictions (Koelsch et al., 2019), can also be quantified as the expected value of the information, or entropy (see, e.g., Hansen and Pearce, 2014). For concision, Table 3 lists some of the central assumptions we will adopt, and provides references to the methods and measures used to implement information theory in our framework.

Computational models have been successfully used to manipulate and analyse the information dynamics of sequences (e.g., Hansen et al., 2021). Most such models are probabilistic: they can capture multiple streams of musical features (see Table 3), and relying on the Markov assumption (see Rohrmeier and Koelsch, 2012), they predict local dependencies. However, predictions for musical and linguistic sequences can span more than just the next event, especially when syntax or harmonic schemas are considered (Rohrmeier and Koelsch, 2012), indicating the need for hierarchical processing across multiple related time scales (see Zuidema et al., 2018). As long as predictions for events with given probabilities are generated these can in principle be used for measuring information and entropy. Our framework will be discussed based on the prediction of the next, discrete event in a sequence, acknowledging that specific models and measures will need to take long-distance dependencies into account.

With these preliminaries in hand, we now turn to a more detailed consideration of the three axes of our framework, applying them to both prototypical song or speech, but also considering atypical or intermediate cases like poetry.

## The ‘Goal’ Dimension: Propositional-Aesthetic Differences

The goal dimension concerns the broader purpose of linguistic or musical sequence productions, whether to convey semantic messages, or to elicit and modulate aesthetic responses in a broad sense (including emotional appraisal, pleasure, movement expressiveness, etc., see Huron, 2016). Both poles of this continuum involve predictions at multiple levels, but the poles differ in how the levels interact.

### Propositionality in Language

The main goal of linguistic acts is arguably to convey propositional meaning: they enable a comprehender to infer the message the speaker intends to convey (Seifert et al., 2013; Kuperberg and Jaeger, 2016). Although speech acts can often convey social relationship, status, sex, origin, etc., paralinguistically (Ladd, 2014), propositionality is nonetheless at the core of language. From an information theoretical perspective this entails reduction of uncertainty about the propositional content transmitted using the current context.

Applying a framework of reverse-engineering and information theory to language, Mahowald et al. (2020) argue that word length, word frequencies, and sequences of phonemes are all designed to optimise the lexicon in order to efficiently communicate, by optimally balancing complexity and informativity. This holds true over a wide variety of languages, and involves tight interactions between multiple linguistic levels. Using a comprehension model that implements both linguistic experience and world knowledge, Venhuizen et al. (2019) showed that entropy reduction is high in propositional words (reducing uncertainty in meaning), and surprisal (information) decreases towards the end of sentences, when the intended message becomes incrementally clearer. However, linguistic sequences involve multiple levels of representation (semantic, syntactic, phonological, etc.), and prediction takes place at all levels (Levinson and Torreira, 2015; Kuperberg and Jaeger, 2016 for a multimodal perspective see Holler and Levinson, 2019; for a critical review see Huettig and Mani, 2016). These levels have also been shown to interact. The hypothesis of uniform information density of a communicative act suggests a constant information rate per unit time (see, e.g., Aylett and Turk, 2004; Piantadosi et al., 2011), and studies show that speakers can actively manipulate information rate at different levels by altering for example phonetic cues, syntactic cues or word length (Mahowald et al., 2013). Specifically, enhanced prosodic prominence or longer durations are used when syllables cannot be predicted well (that is when entropy is high) based on syntactic, semantic or pragmatic contexts (Aylett and Turk, 2004). Comprehenders also use the current context for disambiguation to infer the conveyed message, and higher predictability given current contextual information yields shorter word lengths (Piantadosi et al., 2012; Gibson et al., 2019). This body of language research shows the direct interaction of information and acoustic features given a propositional goal, but also illustrates how conversational situations can be naturally implemented in an information-theoretic framework.

Thus, it appears that propositionality, specifically prediction and inference of encoded messages, profoundly affects the design of languages. The meaning-bearing level is of primary importance, and variations in predictability at the propositional level are balanced by changes in elements within non-propositional supporting levels, like phonology and word choice. These elements vary to enhance predictability (e.g., from context) or to alter the information rate (e.g., by changing in duration), supporting successful decoding of the propositional message. Thus, part of the attested prosodic variability of speech, e.g., in syllable duration or voice pitch, is an effective response that allows variable rates of information and predictability at lower levels, in support of the propositional goal.

### Aesthetics and Reward in Music

Key components of the human reward system relate to prediction (expectancy) and surprise (expectancy violation; Schultz et al., 1997). When an outcome is better than expected, dopamine release is increased, resulting in positive emotions and supporting positive reinforcement learning. Worse than expected outcomes lead to decreased dopaminergic firing, negative emotions and learned avoidance. Dopaminergic firing also predicts the timing of rewarding events (Hollerman and Schultz, 1998). The difference between expected and actual outcomes is termed reward prediction error (Schultz, 2017), and involves predictions about how rewarding a future event will be, as distinguished from sensory predictions about which event will occur (de Fleurian et al., 2019; Koelsch et al., 2019). The extent to which these two predictive contexts—reward prediction error and sensory prediction error—provide appropriate explanatory frameworks for musical pleasure is debated (Colombo and Wright, 2017; Hansen et al., 2017; de Fleurian et al., 2019), but fundamental to either account is the ability to make predictions regarding sequences of sonic events. This ongoing or ‘on-line’ predictive processing is reflected in many theories of musical meaning based on tension-relaxation dynamics (e.g., Meyer, 1956; Narmour, 1990; Huron, 2006; Lerdahl and Krumhansl, 2007; see also Rohrmeier and Koelsch, 2012). However, we note that not all kinds of music rely on expectancy dynamics in order to fulfil their purposes (e.g., Musique concrète).

What design features allow a sequence of sounds to generate expectations, hence to be predictable, but also allow pleasant surprises and (reward) prediction errors? To generate expectations, there must be stable probabilistic relations between elements of a sound sequence, so the probability of particular events occurring concurrently or adjacent to another sound should be higher than random chance levels. Thus, regularity extraction is the foundation of statistical learning in music (Temperley, 2007), and if these relations span multiple time scales, a hierarchical structure of relations can occur (Rohrmeier and Koelsch, 2012; Rohrmeier et al., 2015).

Learned regularities regarding the temporal and spectral relations between events enable probabilistic expectations about which events are likely to occur when (Temperley, 2007). Musical pleasure has been shown to be highest when either prospective uncertainty is low and retrospective surprise is high, or vice versa (Cheung et al., 2019). Since, in music, no one level of the signal is primary *per se* (no single meaning-bearing level of the signal must be unambiguously inferred by a receiver), elements at different levels (e.g., tone frequencies, durational patterns, motifs, etc.) are not constrained to support any one primary level. Thus, both uncertainty and surprise can vary independently of each other at multiple levels, and fulfilment of predictions and surprise can occur concurrently (Rohrmeier and Koelsch, 2012)—think of a certain melodic motif where the expected last tone occurs at the expected time, but within a different harmonic context. This less constrained design allows music to exploit the human reward system very effectively, supporting predictability at some levels and pleasant surprises at others (Zatorre, 2018).

How then can pleasure be gained from repetitive encounters with the same musical piece? Salimpoor et al. (2011) found that for familiar, liked musical pieces, dopamine is released in the striatum both in response to expectations of peak-pleasure events, and to the peak-pleasure events themselves, but in different striatal subregions. This partly explains why, even under low surprise conditions, pleasure can be gained from musical expectations being fulfilled. Representations of musical features might be sparse and decline over time, such that upon repeated listenings new predictions and prediction errors can be generated (Salimpoor et al., 2015). Furthermore, familiar music may remain rewarding upon repeated hearings if its structure is surprising in relation to other pieces of the same genre, that is when it deviates from schema-like representations (Zatorre, personal communication; Salimpoor et al., 2015). Similarly, liking familiar music can even go as far as disliking variant versions of the same song. Repeated listening to a musical piece can also allow listeners to redirect attention to levels not previously attended to and thus to discover new relations between events, again supporting novelty and surprise even in a highly familiar context (Margulis, 2014). Such attentional shifts allow music to occupy a highly rewarding sweet spot between fulfilling the prediction entirely and a total mismatch (i.e., too much information/surprise, see Zatorre, 2018).

In summary, music prototypically enables fulfilment of aesthetic goals while maintaining predictability by preserving the independence of multiple levels of the sound sequence, allowing concurrent surprise and fulfilment of predictions, as well as independent variation of prospective uncertainty and retrospective surprise. Thus, musical design solutions effectively exploit the basic mammalian dopaminergic reward system (Blood and Zatorre, 2001; Ferreri et al., 2019). Hierarchical relations between sounds in a sequence generate expectations in both music and language, but the aesthetic goal alone does not fully explain why particular design features of music arise. This becomes clearer when looking at atypical examples of language and music.

### Aesthetics in Language and Propositionality in Music

Unless lyrics are present (implying a meaning-bearing linguistic layer), music rarely conveys propositional meaning. Exceptions include melodies that themselves stand for messages (e.g., whistling ‘Happy Birthday’ could convey the message of pleasant birthday wishes), ‘songlines’ that encode pathways across landscapes, connected to mythological stories (e.g., by Australian native peoples, Chatwin, 1987), or music that imitates natural sounds (e.g., birdsong). Whistled speech or ‘drum languages’ (cf. Busnel and Classe, 1976) encode propositional meaning in a superficial form, for example using pitch as a replacement of formants or phonemic tone from spoken language.

In such cases, propositional content is woven into the musical structure, and we would expect that exact repeatability plays a crucial role, because surprises would increase the uncertainty of the conveyed meaning. Altering the rhythm of ‘Happy Birthday’ substantially will make it unrecognisable, and keeping the melodic contour but changing the intervals will make it disconcerting or irritating. Imagine someone playing ‘Happy Birthday’ to you in a minor key—would you perceive this as sarcastic or ironic? It seems that in cases of propositionality in music, the acceptable variability of the musical structures is reduced, even more than in speech acts, because here the propositional message is encoded in several levels of the whole musical structure (e.g., pitch and rhythm), not primarily at a single semantic level. Such propositional musical pieces are thus more similar to words than sentences. On the other hand, adding a surprising context could make the piece aesthetically more interesting, thus shifting the goal toward the aesthetic pole.

What is predicted when language is deployed in a mainly aesthetic context? Language can also exploit the human reward system *via* generation of expectations, *via* its hierarchical structure of elements. When the goal is propositional, variations in semantic predictability are balanced by changes of elements within non-propositional levels to maintain a roughly uniform information density (see above). Thus, prospective prediction and retrospective information are tightly linked. But with an aesthetic goal, this constraint can be released, with levels of the signal becoming more independent. Enhancing the predictability of content words is no longer necessary, more variability in predictive uncertainty and surprise become possible, and attention can be focussed on other levels of the sequence. For example, in poetry intonation, phonology (rhyme), durations, stress patterns, etc., appear to vary more independently of propositional content. Propositional content is often not straightforward in poetry, and ambiguity and multiple possible interpretations are frequent. Indeed, some poetry in art movements like Dada, such as Kurt Schwitters ‘Ur-Sonata’ (see Schwitters, 1973), focusses on sound quality rather than propositional content (despite, in historical context, ‘conveying the message’ of ignoring artistic bourgeois conventions). Re-reading or re-hearing a poem can also yield new ways of interpretation similar to re-listening to a musical piece (but see Margulis, 2014). Increased independence of hierarchical levels might allow greater embodiment and/or a more musical perception, for example in a Shakespearean sonnet versus rap.

Infant-directed speech is another example of speech moving toward the aesthetic pole (e.g., Thiessen et al., 2005), although distress in young children is reduced more in response to infant-directed song than infant-directed speech (Corbeil et al., 2016), even for unfamiliar songs (Cirelli and Trehub, 2020). This might be related to the discreteness (high predictability) of pitch and especially duration in music. Our conception of flexibility along the propositional-aesthetic dimension could readily be applied to theatre and opera, both of which have to fulfil both propositional and aesthetic goals concurrently. We predict that predictability is traded off such that passages perceived as highly aesthetic are lower in information content, and vice versa.

To sum up, both language and music can be deployed in atypical propositional and aesthetic contexts, and similar responses follow: with more propositional goals, the multiple levels of the speech or musical sequence are more interdependent, and vary their information density to support successful inference of propositional content. For aesthetic goals, independent variation across levels enables more unconstrained variation in uncertainty and surprise, effectively exploiting the human reward system. However, given that music and speech can both be deployed in the nontypical context, aesthetic versus propositional goals alone cannot explain why certain design features characterize most music (e.g., discrete pitches or isochronous meter) but not speech (e.g., gliding intonation and variable syllable durations). This implies that further dimensions are necessary to explain these design differences.

## The Novelty and Repetition Dimension

The novelty-repetition dimension is closely linked to the propositional-aesthetic dimension. This dimension involves the repeatability of elements and their relations at different scales (from single elements to entire pieces) and at multiple levels of musical or linguistic sequences, and their balance in use with novel elements and relations. Generally, repetition enhances predictability, whereas novelty is unpredictable and thus high in information.

### Repetition in Music

One of the design features distinguishing prototypical music from language cross-culturally is that music is characterised by repetition at multiple levels (Fitch, 2006; Savage et al., 2015). Repetition can involve single notes, melodic motifs, chord progressions, rhythmic patterns, and the entire musical piece. Repetitiveness in music seems to be also a foundational perceptual principle: the speech-to-song illusion is a striking phenomenon in psychological research on music and language, whereby repetition of speech phrases leads to them being perceived as sung speech (Deutsch et al., 2011). Certain speech phrases, especially when characterised by relatively flat within-syllable pitch contours and less variability in tempo, are more prone to be judged as musical by Western listeners (Tierney et al., 2018). The repetition effect has recently been generalised to repetitions of random tone sequences (Margulis and Simchy-Gross, 2016) and of environmental sounds. These were judged as more musical by Western listeners (Rowland et al., 2019), suggesting that repetition leads to the inference of structural relationships between repeated sounds (cf. Winkler et al., 2009), which are then cognitively interpreted as ‘musical’.

What specific features of music allow or select for repeatability? Prior to recording technology, repetition entailed that a sound sequence be remembered and reproduced. To be remembered a sequence must be distinguishable from other, similar sequences (e.g., related melodies or rhythmic patterns), and learnable by establishing relationships between the constituent events. The existence of sound categories and hierarchical rules to combine them (Herff et al., 2021; see also Rohrmeier and Pearce, 2018a,b) enables this. The musical design solutions in this respect are discrete tones in scales (in a hierarchical relation), and durations related in a simple fashion. From an information theoretical perspective, this means that the possible uncertainty about forthcoming musical events is reduced from the outset by adopting a smaller ‘alphabet’. This allows a lower number of plausible continuations of a sound sequence than if frequency and temporal dimensions were unconstrained. Because hierarchical relations exist between tones this factor also constrains plausible continuations among distant elements. Reduced alphabet size also supports statistical learning and the application of Gestalt principles, both relevant for prediction in music (Snyder, 2000; Morgan et al., 2019).

Repeatability in music seems to be particularly related to the fact that the temporal dimension in music is also hierarchically structured—durational patterns are related to an underlying meter. First, meter supports embodiment *via* beat extraction and entrainment (Kotz et al., 2018), adding a strong motoric component that may increase the memorability of musical sequences (Brown and Palmer, 2012). Second, meter can also function as a kind of glue between multiple levels of a musical sequence by enforcing relations among them, including higher-order levels like chord progressions, motifs etc. The auditory system is able to make predictions and track deviations at multiple levels at the same time (Vuust et al., 2011). High uncertainty in memory at one level of the musical signal (e.g., in melodic arrangement of pitches) can be countered by low uncertainty in another (e.g., rhythm), reducing the joint uncertainty of both levels and enhancing the confidence in the prediction of the ongoing musical sequence (‘I remember that this particular pitch followed with this rhythm’).

Is repeatability sufficient to explain the occurrence of discrete pitches on scales and meter in music? Rapid learning of auditory events is even possible for arbitrary sounds that are repeated within a stream of random sounds (Agus et al., 2010), suggesting that the auditory system is capable of finding repetition in the auditory stream irrespective of discreteness. This observation is consistent with our claim that specific design solutions for repeatability in music are not strictly necessary for perception, but relate to (re-)production. However, humans are easily capable of reproducing sound sequences that are not characterised by a reduced alphabet in the frequency and/or temporal domain. This suggests that repeatability is not a sufficient explanation for these design solutions of music. What seems to be crucial, we will argue below, is the interactivity between individuals in a group, when making music together in a choric context.

To summarise, repeatability in musical performances involves a reduction in the alphabet in multiple dimensions. This enables higher predictability and structural relations in a hierarchical manner between elements. In music, meter allows strong temporal predictions, enforcing predictive relations in higher-order levels and enabling a strong link to motoric processing. Scales in melody allow equally strong frequency predictions, since the pitch of possible following notes is strictly circumscribed.

### Novelty in Language

As emphasised above, language is mainly concerned with the primary goal of transmitting propositional meaning. These messages conveyed should be relevant and informative, and thus (typically) novel (Grice, 1975; Sperber and Wilson, 1986). The novelty typifying language acts is therefore closely linked to propositionality. What design features enable novelty in language? Crucially, language is characterised by duality of patterning (Hockett, 1960), and can be analysed both as an arrangement of meaning-bearing units (morphemes and words) supported by a lower-level arrangement of meaningless phonemes. Meaning-bearing units can be rearranged to convey new messages, which is termed productivity (Ladd, 2014). This productive layer is the main one that realises novelty (although neologisms can also enable novelty at the phonological level). Even repetition of propositional content is typically realised by a different arrangement of words or morphemes.

In language, repetition as a structural relationship of (relatively) categorical sound elements does occur at the phonemic level, where learned structural relationships between phonemes hold within a particular language. This is comparable to reduction of sound categories in music: a finite set of phonemes and specific restrictions on their combinations reduces the uncertainty of which phoneme could follow in a sequence. Words are also repeated (although the size of the lexicon is vast). Indeed, long-term memory for melodies has been proposed to be comparable to the word lexicon (Peretz et al., 2009). Language therefore can be interpreted as balancing novelty and repetition, prototypically by differentially deploying them at different levels of the linguistic stream—phonological repeatability enables morphosyntactic and semantic novelty. Thus, in prototypical conversational language, novelty is realized at the morphosyntactic and semantic levels, with phonology and the rote-memory lexicon playing a supporting role.

### Repetition in Language and Novelty in Music

What happens when repetition in language occurs at the productive level, that is with morphemes, words, and sentences? Some instances of repetition are relevant in a propositional sense: repetition of the same word or morpheme (reduplication) can be used for emphasis, or serve grammatical functions like plural marking (Hurch and Mattes, 2005). Repetition might also encourage the receiver to seek different interpretations of the phrase that are not apparent at the first glance, to resolve ambiguity (Knox, 1994).

Some situations however require the repetition of entire speech phrases, for example in ritualised contexts. When memorability needs to be enhanced, this is achieved by emphasising structural relationships in other levels of the speech phrase like intonation, stress, using rhyme or specific repeated syllabic patterns (e.g., poetic forms). This can also be observed in infant-directed speech which is very repetitive (Margulis, 2014). A link to memory might be that attention allocation seems to be related to surprising events (Forth et al., 2016; Koelsch et al., 2019). In the event-related potential, a mismatch negativity, indexing unpredicted and thus surprising events, is usually followed by a P3a component, associated with attention allocation (Schröger et al., 2015). More independence of levels of the speech signal would enable more surprising events due to possible unexpected interactions between levels, emphasising the structural relationships between them. On the other hand, predictive cues can also guide attention to a specific stimulus or stimulus feature (Gazzaley and Nobre, 2012), enhancing memory encoding. Our framework predicts that actions with an aesthetic goal, where we expect a greater independence of representational levels of the sequence and more variety in predictability, should be remembered better. In line with this, Margulis (2014) proposes that memory for music, poetry or utterances with schematic form, like jokes, is based more on acoustic surface structure than in conversational speech: speech involves attention allocation towards propositional content. Note that this enables paraphrasing the same propositional content with different words, which is more difficult for musical structure with notes or chords.

Turning to novelty, because attention is drawn to surprising events (Forth et al., 2016; Koelsch et al., 2019), listening to music that is highly predictable and unsurprising could lead to attentional shift and boredom. Thus, an additional pressure for music is to include a degree of novelty. One design solution to balance both novelty and repetition is meter (hierarchical relation of durational patterns relative to a beat). Meter provides a predictive framework within which novelty—unexpected and surprising events—is well defined (e.g., syncopation). Because multiple levels of the signal allow for predictability within and across levels by means of probabilistic relationships between their elements (tones, intervals, chord progressions, etc.), each level also allows for surprise. In repeated performances novelty can be provided by slight shifts in performance style, tempo, expression, etc., making the interpretation of familiar pieces a common focus of Western classical music concerts or opera. Concerning recordings, the possibility of attentional allocation to different levels of the piece with each repeated listening could be interpreted as listener-generated ‘novelty’, since new, potentially surprising, relations might be perceived. Thus, music also balances novelty and repetition in multiple ways, but they are quite distinct from prototypical conversational language.

In summary, both music and language balance repetition and novelty, but in different ways. While language usually allocates repeatability to the phonological level and novelty occurs at the morphosyntactic and semantic levels (related to propositionality), music typically allows both novelty and repeatability across all levels of the musical sequence, and meter seems to be especially crucial as a predictive layer throughout, enabling both prediction and surprise. However, language can also be repeatable at the word and sentence level. Thus, despite the clear validity and value of the two traditional dimensions on which music and language are differentiated—goal and novelty—certain design features are still not fully explained. We therefore suggest that understanding the design differences of language and music require a further explanatory dimension, to which we now turn.

## Interactivity: The Choric-Dialogic Dimension

Our proposed interactivity dimension concerns the temporal coordination of linguistic or musical productions of multiple participants. Both concurrent production, in choric mode, and dialogic turn-taking involve joint actions that are causally coupled, but they pose different constraints on predictability in sequences.

### Dialogic Contexts in Speech

Two speakers talking at the same time constitute noise for each others’ speech signals: overlapping signals make propositional content harder to decode (Fargier and Laganaro, 2019). Therefore (among possible roots in cooperative social interaction, see Levinson, 2016; Pika et al., 2018; Pougnault et al., 2022; but see Ravignani et al., 2019), dialogic contexts favour the avoidance of overlap and the coordination of turn-taking behaviour (Levinson and Torreira, 2015; Levinson, 2016), and signals should be designed such that receivers can predict the ending of an utterance (see, e.g., Castellucci et al., in press). Information should therefore be low (and predictability high) at the end of signal sequences. Given the requirement to reduce uncertainty in conveyed propositional messages, this should lead to high information density during most of the signal sequence (to optimally exploit the speech channel capacity, see above), with a decrease in information towards its end.

In dialogue, turn completions seem to be predicted based on both prosodic cues (Bögels and Torreira, 2015) and lexicosyntactic content (de Ruiter et al., 2006; Torreira et al., 2015), whereby semantic content seems to be more important in predicting the end of a speaker’s phrase than syntax (Riest et al., 2015; see Jongman, 2021, for an overview). On the other hand, content prediction might be used to enable early response planning in parallel with comprehension of the current turn (Levinson and Torreira, 2015; Corps et al., 2018), which helps to avoid large gaps between turns that could themselves be interpreted as meaningful (e.g., Pomerantz and Heritage, 2013). Accordingly, Castellucci et al. (in press) proposed separate pathways for turn-timing and response planning. Since response planning requires neural resources that might compete with those utilised for comprehension (Bögels et al., 2015, 2018, see also Knudsen et al., 2020 for the role of backchannels, fillers and particles in this regard), it should start once the semantic uncertainty is low enough, and preferentially happen in places along the sequence that are low in information. Once the remainder of the sequence is highly predictable, interlocutors can exchange the roles of sender and receiver: taking turns. The next utterance will again start high in uncertainty, requiring informative events, until it nears its end.

The information density trajectory must be perceivable if the current receiver is to be able to predict when uncertainty is low enough to take the floor. This requires an ongoing monitoring of the information density in the received signal and a continuous prediction of the amount of uncertainty reduction that will follow from later events. That is, listeners need to predict when new events do not reduce uncertainty much further, probably based on the semantic uncertainty of the conveyed message and taking several past events into account to capture the general information trajectory. If the time point of turn-taking is marked by low information density, then this should be unambiguously distinguishable from local information density minima that may occur in the signal before. In the case of language, some words are more informative than others even when the speech utterance is not finished yet (Venhuizen et al., 2019). Because a speech act consists of multiple interacting and integrated levels—phoneme level, morpheme and word level, prosodic intonation, stress, lexical tone, etc., as well as paralinguistic information like facial or body expressions (see e.g., Holler et al., 2018; Wohltjen and Wheatley, 2021) and contextual cues in the environment (Ladd, 2014; Kuperberg and Jaeger, 2016; Holler and Levinson, 2019), each level can play its own part in reducing uncertainty about the propositional semantic content that should be conveyed. In line with this, phrase-final lengthening (a prosodic cue that can signal turn-ending, see Wightman et al., 1992) would decrease information per unit time, while in turn speakers accelerate their speech rate and thus information per unit time when they want to continue their utterance (Walker, 2010). We would predict that in order to mark the ending of a speech phrase and to take turns, the end of a speech phrase should be highly predictable across all levels of the sequence, even if one feature (like falling intonation) might preferentially mark the ending of the speech phrase at a prosodic level (see de Ruiter et al., 2006). Evidence seems to confirm that prosodic, lexical and syntactic levels interact to mark turn-taking (reviewed in Forth et al., 2016). This makes sense: if only one single level, such as falling intonation or lengthening, predicted the end of the current speaker’s utterance, it would be highly surprising if an unpredicted, highly informative event occurred at another level (for example a highly surprising word). Information density would locally peak and the receiver would likely stop their preparation to take turns and re-allocate attention. Precisely this cross-level effect is used in investigations of speech processing by event-related potentials such as the N400, an evoked potential component which deflects negatively when target words in sentences are semantically unexpected (e.g., Grisoni et al., 2017; but see Maess et al., 2016 for a differential effect for verbs and nouns), even if there is no surprise at all other levels of the speech stream (e.g., intonation, syntax, phonology, etc.). Syntactic violations in contrast are indexed by earlier evoked components like the ELAN (Hahne and Friederici, 1999), illustrating that the brain uses prediction at multiple levels of the speech stream in parallel.

The example mentioned above also illustrates an interaction with the propositional-aesthetic dimension. If there is propositional content, the representational levels of the sequence that carry this content are of primary importance, while other levels support the semantic predictability and are thus less free to vary in their information trajectories than when propositional content is absent (as in nonsense speech or music). An event high enough in information at a supporting level might both alter the semantic understanding and disturb the turn-taking process. Thus, robust semantic understanding under the constraint of efficiency (Gibson et al., 2019) might facilitate successful turn-taking as well. In contrast, the less focus is on the propositional content, the less a need for hierarchy among levels exists, which means information density among levels should be freer to vary, possibly converging only towards the end of the signal sequence to enable turn-taking.

Interestingly, if there are more than two participants in the conversation, information density alone cannot be used to coordinate who will start the next speech act. In order to achieve this, paralinguistic information like pointing gestures, naming or rules about who speaks next need to apply (e.g., Mondada, 2013). The empirical prediction would be that the larger the group, the higher the danger of overlap between former receivers’ initiation of speech acts, and the more the requirement for paralinguistic coordination (or an individual designated to choose the next speaker, e.g., the chair of a meeting). However, such overlap should occur only after one interlocutor ends their speech phrase, since all receivers can predict the end of the current speaker’s turn.

To sum up, dialogic contexts require that endings of sequences are perceivable by a decrease in information density (which means an increase in predictability) across levels of the sequence, such that later events have on average lower information than former events. Both language and music are designed to fulfil this requirement in dialogic contexts. The propositional focus in most spoken dialogues adds an additional constraint that non-propositional levels should be subordinate to the levels conveying propositional content.

### Choric Contexts in Music

Turning to the concurrent, choric pole, successful concurrent performance requires that signals do not disrupt processing when they overlap. One design feature that avoids masking by concurrent sounds (which is more effective when frequencies are more similar, see Moore, 2014) is to make them discrete and related by small integer ratios, as are the tones on musical scales. One example is the octave, whose existence across cultures is a statistical universal, and which enables all members of a group to sing in unison even when males’ vocal range lowers after pubertal vocal change (Harries et al., 1998). In line with this, octave equivalence (perceiving two pitches as categorically the same when they are an octave apart) seems not to be perceived in a culture where individuals rarely sing together (Jacoby et al., 2019). This ‘simple ratios’ constraint interacts with another melodic design feature: reduction of the set of possible tones by limiting them to a small set (a ‘scale’). Again, in information theoretic terms, establishing pitches on scales with strict tonal relations involves a reduction of the alphabet of allowed symbols along the fundamental frequency dimension (for a proposal for the roots of tonality in the physiology of hearing see Trainor, 2018). This limited set of possible tones allows individuals to join a music making chorus, match the produced sound sequences and/or complement them (for example in BaAka polyphonic singing, see Lewis, 2021), thus contributing to a unified sound entity in a coherent performance (a joint action with the deliberate coordination of actions, see Sebanz and Knoblich, 2009, 2021; Tomasello, 2010; Keller et al., 2014) rather than generating a set of sounds that are not causally coupled (cf. Ravignani et al., 2014). If scale tones are hierarchically related, the continuation of a melody can be predicted with a limited uncertainty by the participating individuals, allowing them to contribute in an ongoing manner, as well as allowing for variation and thus individuality in their contribution (cf. Savage et al., 2021).

The choric context also requires that events in separate sound streams should be tightly coordinated in time. Uncertainty in timing would lead to disintegration of concurrency and coordinated joint action. Therefore, the signal should be designed to enable high-precision temporal predictability throughout. One key design feature that enables such predictability is isochrony. Tight coordination however requires participants to attend to the other participants’ actions, since ongoing coordination means prediction and monitoring on a moment-to-moment basis (see, e.g., Keller et al., 2014). If the next element can be precisely predicted in time, then the temporal information gain from each event is low, which lowers attentional demands (Koelsch et al., 2019). On the other hand, unpredicted events capture attention. How can these two requirements—high predictability and ongoing attention allocation—be aligned? If isochrony happens not at the level of each individual event, but on a meta-level, providing a scaffolding which still enables novelty (see the section about the novelty dimension), then both requirements can be fulfilled. The design solution satisfying these constraints is the concept of hierarchical meter, which allows certain placements in time and forbids others, but which also gives room for variability to create novelty since not each possible slot needs to be filled by an event. Again, meter represents a reduction in the alphabet, in this case a small set of possible onset and duration patterns relative to the beat.

Unlike in spoken language, there is much less noise and thus much less uncertainty when different participants in a choric performance contribute with different events to each of the levels of the musical sequence. We would therefore expect more degrees of freedom in terms of what event—which tone or chord—occurs than in speech deployed in a choric context (see below). However, the timing, that is when events occur in choric performances, is crucial and needs to be coordinated in a precise manner. Note that with a meter, events do not need to be played all at the same time (synchrony) nor be evenly spaced in time (isochrony). Rather, the burden is to keep events within the metrical scaffolding, so that the contributions of the participants relate to each other in the moment, or there would no longer be one coherent performance. Even in cases of notated music where it is clear which note must be played when, there is still the need for coordination among musicians, and in the absence of strict isochrony, a coherent performance needs to be otherwise synchronised, for example by using participants’ body motion.

In summary, we argue that meter is a crucial design feature that develops in music deployed in a choric context, with the goal of balancing coordination and attention, while still permitting variation or improvisation. Meter provides a predictive fabric throughout an ongoing performance. This is complemented by a reduction of the alphabet in the tonal domain: a limited set of hierarchically related pitches allows accurate predictions of possible continuations of an ongoing acoustic performance, and of multiple different complementary event streams, without compromising the coherence of the overall performance as a joint action.

### Dialogic Contexts in Music and Choric Contexts in Speech

Dialogic contexts also can occur in music, for example when several musicians take turns soloing in jazz, or in call-and-response singing. Here the same constraints must apply as for spoken dialogue, and we expect musical phrases to show lower information density towards the end. Thus, there must be a means to increase predictability at phrase endings. This aligns well with the notion of musical closure in harmony and melody, or the tendency of melodies to be shaped like an arch (Huron, 2006). Again, in music we expect that phrase endings tend to have low information density across all levels of the sequence on average. There are conventions which time or mark musical phrase endings, like the number of beats a soloist has available to perform their solo, or certain rhythmical or musical motifs, but we predict that even in these cases there should be decreasing information density towards musical phrase endings. An example illustrating how the prediction of a phrase ending can be disturbed if one level is high in information, at the level of harmony, are deceptive cadences in Western classical music, where a surprise chord replaces the highly predicted tonic as final chord, disrupting an expected sense of closure.

Again, we would not expect that one signal level is primary over another, at least if there is no propositional content (as typical in music). Rather, we expect that the levels of the sequence have more degrees of freedom to vary in their information content, as long as the phrase ending remains predictable. For example, a soloist in jazz might introduce a harmonic modulation (with high information content at the harmonic signal level) at the end of a phrase, while keeping melodic and rhythmic levels highly predictable and thus inviting a turn-taking event after which the next solo now occurs in a new key. That musical phrases in a more general sense exist, for example phrases in a solo Lied (song), could thus be an abstraction of deployment in a dialogic context.

What design features are predicted for language in a choric context? The particular tendency of overlapping speech stimuli to act as each other’s noise (thus increasing uncertainty) means that simultaneity can only occur if precisely the same words or syllables are uttered at the same time, as happens for example in simultaneous speaking in religious or theatre performances (chanting). The prediction here is that attention is much more focussed on coordination than in dialogic speech acts, and that an isochronic and/or metrical scaffolding should develop (cf. Bowling et al., 2013). Since word order in such a sequence must be pregiven, and thus the information density of the word and phoneme level would be constant and very low, we would expect suprasegmental or paralinguistic levels to vary more in information density and to be used to reduce uncertainty regarding timing. That means body motion, facial expressions or prosodic intonation should be more pronounced in a spoken choric context.

## Comparative Perspective

We suggest that the perspective of deployment of sound sequences in a three-dimensional quality space (goal: propositional-aesthetic, novelty: repetition-novelty, interactivity: dialogic-choric) along with the information theoretic concept of reducing uncertainty can also be used in bioacoustics research. We are aware that transferring a concept directly from human language and music might not work for animal communication, but we think that, especially for complex vocal displays in birds or whales, our framework may provide some insight, since these ‘animal songs’ bear some structural similarities to language and music (Rohrmeier et al., 2015). Information theory has been employed in animal communication research, although the term ‘information’ has often been used in a colloquial sense or inconsistently (Stegmann, 2013; Fitch, 2014). We hope that applying our framework provides some useful insights regarding complex vocal displays, along with call combinations and sequences (Engesser and Townsend, 2019). The information theoretic framework also encourages us to consider non-vocal levels like body motion that might be especially relevant for mating displays (Mitoyen et al., 2019).

When talking about ‘goals’ in animal communication, it is necessary to consider that goals other than propositional or aesthetic ones (e.g., social bonding by vocal convergence, providing information about sex or status, etc.) might use the vocal domain independently of the dimensions we derived for human language and music. Often it is unclear what animals communicate in their vocalisations, and some researchers question the existence of communicative content at all, proposing instead that animals manipulate others by means of their signals (Owren et al., 2010; Stegmann, 2013). On the other hand, there is evidence in some cases that animal calls can be functionally referential, reliably co-occurring with external entities (Seyfarth et al., 1980; Price et al., 2015), but little evidence that complex vocalisations like bird song or whale song have functional referential meaning (Engesser and Townsend, 2019). Analysing information trajectories across multiple levels of the sequence might give additional insight into this important question, but this requires that several such levels can be disentangled in the first place, which might not be easily the case in animal vocalisations.

Duality of patterning appears to exist only in human language (Bowling and Fitch, 2015). When vocalisations have a propositional goal (in the sense of referring to external entities and eliciting reliable behavioural responses), we would therefore expect this content to be encoded in a whole structure across levels of the communicative signal, similar to music when deployed in a propositional context. We would also expect high predictability within the signal (since surprising elements could potentially add uncertainty to the inferred content) and probably a relatively uniform information density to enhance transmission. In turn, given that reward systems in other animals (Connell and Hofmann, 2011) would also be related to prediction and surprise (Schultz, 2016), we would expect that sound sequences with independence in information variation between different levels are more likely to fulfil an aesthetic rather than a propositional goal. We speculate that, in mating displays, constrained surprise rather than complexity is what makes displaying individuals attractive for mating, accounting for the common occurrence of individually distinctive songs and song repertoires.

Animal vocalisations like bird or whale song consist of subunits that can be structured in a hierarchical manner, and thus bear some structural similarities to human language and music (Payne and McVay, 1971; Rohrmeier et al., 2015). Repetition of subunits and their recombination often characterise such complex vocal displays. We would expect that subunits used in repetition are categorical to reduce the alphabet and enhance predictability. We would also expect a tendency towards hierarchical organisation in the temporal domain for longer vocalisations relative to shorter ones within comparable species.

What about novelty? Novelty might be realised by using new sounds with high surprisal. For example, the best documented example of ‘vocal’ learning in chimpanzees involved the addition of a lip buzz or ‘raspberry’ at the end of pant-hoot sequences (Marshall et al., 1999). However, since such new sounds would not reduce uncertainty in decoding a message for the receiver, unless an association to some external entity is learned, they would probably not have ‘content’ in the sense of functional referentiality. Novelty can also be realised by rearranging subunits that are structurally highly predictable, as is often characteristic of bird song (Kroodsma, 1978) and somewhat structurally similar to human song (Lomax, 1968).

Repetition can also have referential relevance in call combinations or sequences (see Engesser and Townsend, 2019), for example in chickadee call repeats (e.g., Hailman and Ficken, 1987). Some of these calls appear to be categorical (in the sense of being discriminable) while others are higher in variability. Moreover, some of these combinations are rather short. It seems that such call combinations may not be straightforwardly accounted for by our three-dimensional framework. However, we expect that for stand-alone calls, uncertainty should be higher than when another call (same or different) is appended, as evaluated by changes in attention of receivers. Calls that occur only in fixed combinations might however not induce surprise since there is little uncertainty in referential content when encountering them.

Using the predictions derived from deployment of human language and music in choric or dialogic contexts could reveal whether animal vocalisations show design features based on differential temporal coordination of signals (Ravignani et al., 2019). Coordinated vocal displays, both concurrent and turn-taking, are widespread in animal communication. Duetting for example is widely observed in bird species. Investigating the information trajectories could reveal whether and how individuals relate to each other in their vocalisations. Interlocking vocalisations between two pair-bonded or courting birds for example could be investigated to see whether it is based on decreasing information density after one phrase. This would indicate a dialogic deployment. Competitive vocal displays, for example between two males in territorial contexts, might show dialogic design features with decreasing information density at the end of an individual’s vocal phrase. If the display involves masking the competitor, we would predict that overlap is done in moments of high information. On the other hand, duets might be based on a coordination of each event with predictability enabled by some isochronous scaffolding, similar to a musical piece where performers do not play each note concurrently but nonetheless contribute to a unified musical piece. Such ‘hocketting’ would be indicative of a choric deployment. Bird species differ in their preference for overlap or overlap avoidance and in their flexibility depending on social and environmental context (Pika et al., 2018). Starlings seem to be a particularly interesting model species, showing both turn-taking and overlapping vocalisations depending on social context, and varying in their proportion of either by sociality of subspecies (Henry et al., 2015). We would predict that in all these cases information trajectories are perceived by receivers and used to coordinate their own vocalisation in relation to that of the other individual(s).

Other vocal displays that might be interesting to investigate in this context are antiphonal calling in elephants (Soltis et al., 2005) or bats (Carter et al., 2008) or duets in gibbons (Geissmann, 2002) or indris (Gamba et al., 2014), as well as group calling in meerkats (Demartsev et al., 2018). Castellucci et al. (in press) suggest singing mice as a model species for coordinated vocal timing. Bottlenose dolphins can switch between alternating vocalisations and simultaneous duetting (Lilly and Miller, 1961). We would predict that their alternating vocalisations are coordinated by decrease in information density at the end of phrases, while their duetting might be coordinated on an event-by-event basis as in choric deployment. A particularly interesting case is the group territorial display of plain-tailed wrens (*Thryothorus euophrys*) where group choruses including multiple males and multiple females are used to jointly defend a territory. In this species simultaneous performance occurs within sexes, with turn-taking between sexes (Mann et al., 2006) within a chorus. We expect within-sex performance to be characterised by a tendency toward an isochronous scaffolding, comparable to meter, and turn-taking between sexes to be coordinated by terminal decreases in information density.

Animal group communicative (choric) displays often appear uncoordinated, for example in howler monkeys (Sekulic, 1982) or, at the other extreme, highly synchronised, as in some fireflies (see Ravignani et al., 2014). Group coordination can either be based on predictive or reactive behaviour, and we suggest that information trajectories could be examined to address this issue. If individuals in a group (for example howling wolves) vocalise in a coordinated manner, we expect each individual’s contribution to reduce uncertainty about event timing for the other individuals. This would be indicative that the performance aims at creating a coherent entity, implying choric design features.

## Conclusion

In this paper we have proposed a framework based on information theory, adopting a design stance to investigate differences in language and music. We suggested that some key design features of music and language can be explained as responses to their deployment between dialogic and choric poles of a continuum rooted in interactive performative constraints. This interactivity (choric-dialogic) dimension complements the widely recognised goal (propositional-aesthetic) and novelty (repetition-novelty) dimensions, forming a three-dimensional framework within which different forms of music and language can be placed, and their design differences understood.

We argued that the goal and novelty dimensions alone are not sufficient to explain differences in design features between music and language: the interactivity between individuals is crucial. For dialogic contexts, the only coordinative constraint concerns the timing of the turn-taking between individuals, which should be indexed by a lower information density towards the end of phrases, across all levels of the sonic stream. When there is also a propositional goal, non-propositional levels should be constrained in their variability to support the decoding of propositional content. Information rate should be high—realised mainly by novelty at the propositional level—until turn-taking is indicated. Conversational speech acts and turn-taking are the prototypical features that fulfil these requirements.

In contrast, choric performance requires tight temporal coordination of all contributing individuals, as well as avoidance of masking by simultaneous sound events, enabled by high predictability in timing and frequency of sonic events. When there is also a pressure for novelty, isochronous meter and discrete pitches in scales are design solutions that enable a group of participants to join in making a coherent sound sequence, allowing both novelty and repeatability. This contributes to the independence of multiple levels in the sonic stream with regard to surprise and uncertainty, making these independent levels well suited to exploit the human reward system. The prototypical form of choric performance is joint music making, but our framework also encompasses non-canonical forms of music and language like chant, poetry, or exchange of musical solos, thereby avoiding an overly simplistic dichotomy between language and music. Furthermore, our framework supports comparisons of different forms of communication across distinct modalities and species and can help to generate new hypotheses about optimal design of signals satisfying multiple different requirements. We hope that this framework will also be fruitfully employed in animal communication research, broadening the scope of comparisons with music and/or language.

## Author Contributions

All authors listed have made a substantial, direct, and intellectual contribution to the work and approved it for publication.

## Funding

This article was supported by the Austrian Science Fund (FWF) number W1262-B29 (DK Grant Cognition & Communication). FH was also supported by a VDS CoBeNe final fellowship. The funders had no role in decision on the article content, decision to publish, or preparation of the manuscript.

## Conflict of Interest

The authors declare that the research was conducted in the absence of any commercial or financial relationships that could be construed as a potential conflict of interest.

## Publisher’s Note

All claims expressed in this article are solely those of the authors and do not necessarily represent those of their affiliated organizations, or those of the publisher, the editors and the reviewers. Any product that may be evaluated in this article, or claim that may be made by its manufacturer, is not guaranteed or endorsed by the publisher.
